# Hybridization increases invasive knotweed success

**DOI:** 10.1111/eva.12139

**Published:** 2014-01-02

**Authors:** Madalin Parepa, Markus Fischer, Christine Krebs, Oliver Bossdorf

**Affiliations:** 1 Institute of Plant Sciences, University of BernBern, Switzerland; 2 Institute of Evolution and Ecology, University of TübingenTübingen, Germany; 3 CABI Europe SwitzerlandDelémont, Switzerland

**Keywords:** allelopathy, biological invasions, competitive ability, *Fallopia*, hybridization, invasiveness

## Abstract

Hybridization is one of the fundamental mechanisms by which rapid evolution can occur in exotic species. If hybrids show increased vigour, this could significantly contribute to invasion success. Here, we compared the success of the two invasive knotweeds, *Fallopia japonica* and *F. sachalinensis*, and their hybrid, *F. *× *bohemica,* in competing against experimental communities of native plants. Using plant material from multiple clones of each taxon collected across a latitudinal gradient in Central Europe, we found that knotweed hybrids performed significantly better in competition with a native community and that they more strongly reduced the growth of the native plants. One of the parental species, *F. sachalinensis*, regenerated significantly less well from rhizomes, and this difference disappeared if activated carbon was added to the substrate, which suggests allelopathic inhibition of *F. sachalinensis* regeneration by native plants. We found substantial within-taxon variation in competitive success in all knotweed taxa, but variation was generally greatest in the hybrid. Interestingly, there was also significant variation within the genetically uniform *F. japonica*, possibly reflecting epigenetic differences. Our study shows that invasive knotweed hybrids are indeed more competitive than their parents and that hybridization increased the invasiveness of the exotic knotweed complex.

## Introduction

Hybridization is an important and common evolutionary process in plants and animals (Arnold 2004). Despite the potential complications that hybridization can cause for the survival and in particular reproduction of hybrids (Husband 2000; Ramsey and Schemske 2002), their frequency is particularly high in rapidly radiating groups (Abbott et al. 2000; Seehausen 2004; Grant et al. 2005; Mallet 2007), which indicates that hybridization also offers advantages. For instance, novel genetic combinations may enable these taxons to outcompete and eventually displace their parents (Buerkle et al. 2000) or they may provide evolutionary innovations that allow them to cross valleys in the adaptive landscape that limit the adaptation of the parents (Barton 2001; Rieseberg et al. 2003).

Invasive exotic species are particularly interesting in this context, because they offer many well-documented examples of rapid evolution in which hybridization appears to play an important role (Abbott 1992; Ellstrand and Schierenbeck 2000; Zalapa et al. 2010; Blair et al. 2012). Oftentimes, the rapid spread and resulting ecological and economic problems of invasive species only begin after a lag phase (Ewel et al. 1999; Crooks 2005), possibly because these species first undergo evolutionary changes (Sakai et al. 2001; Lee 2002; Bossdorf et al. 2005; Suarez and Tsutsui 2008). For some of these species, these may be inter-or intraspecific hybridization events. There are many cases where hybridization of invasive species has been documented with molecular methods (e.g. Milne and Abbott 2000; Gaskin and Schaal 2003; Gallagher et al. 2011). Previous studies that compared the invasiveness of hybrids to that of their parents found that hybrids can be more plastic and more tolerant to environmental conditions (Weber and D'Antonio 1999), better competitors against native plants (Daehler and Strong 1997) or spreading faster than parents (Vila and D'Antonio 1998; Hovick et al. 2012). However, such direct comparisons of invasive hybrids and their parents remain scarce.

Some of the most important plant invaders of temperate ecosystems are the knotweeds *Fallopia japonica* (2*n* = 88), *F. sachalinensis* (2*n* = 44) and their hybrid *F. × bohemica* (2*n* = 66) (Bailey et al. 2007). Both parental species have been introduced from Eastern Asia to Europe, and later North America, in the 19th century as ornamentals and forage plants (Bailey and Conolly 2000). About 50 years later, the first records of the hybrid of the two knotweed species were documented, and there are currently many different hybrid genotypes spreading in Europe and North America (Mandak et al. 2004; Bailey et al. 2007; Krebs et al. 2010). Initially a tremendous commercial success and very popular garden ornamentals, the species later became very aggressive invaders of ruderal habitats and in particular river banks (Pysek et al. 2009). Invasive knotweeds grow extremely rapidly, form extensive rhizome networks, cause significant changes in the nutrient cycles of invaded ecosystems (Dassonville et al. 2007), and they eventually displace most native plants (Hejda et al. 2009; Aguilera et al. 2010).

A few previous studies suggest that the three knotweed taxa – *Fallopia japonica*,*F. sachalinensis* and *F. × bohemica* – differ in their competitive ability. Some of the hybrid genotypes possess a greater ability to regenerate from rhizome fragments than their parents (Bimova et al. 2003; Pysek et al. 2003). The hybrid is also currently spreading faster than its parents (Mandak et al. 2004), and it is possible that the genetic diversity of hybrids contributes to this (Schnitzler and Bailey 2008; Bailey et al. 2009). However, many of these observations have been made in the field, and we still do not know to what extent the superiority of hybrids is indeed an inherent property versus reflecting different environmental conditions. Another important question is how within-taxon variation of hybrids compares to that of its parents – if this variation is indeed a key determinant of success.

There is increasing evidence that the capacity to exude chemical compounds that are detrimental to native plants (= allelopathy) can contribute to plant invasiveness (Inderjit et al. 2008). Previous studies showed that invasive knotweeds can impact native plants also through allelopathy (Siemens and Blossey 2007; Murrell et al. 2011), and a laboratory experiment suggested that the hybrid may have increased phytotoxic effects on the germination of native species (Moravcova et al. 2011). It is therefore interesting to find whether the hybrid superiority is explained by such belowground chemical interactions.

Here, we conducted a common garden experiment to compare the success of invasive knotweed hybrids and their two parents when competing against a community of native species. We asked the following questions: (i) Do knotweed hybrids show greater regeneration success and/or growth than their parents within a native plant community? (ii) Do hybrids have a greater impact on native plants? (iii) If hybrids and parents differ, does allelopathy play a role in this? (iv) How much intraspecific variation in competitive ability exists among different clones of hybrids versus parental species?

## Methods

### Plant material

For our study, we used plant material from 27 different clones of *Fallopia* × *bohemica*, 13 clones of *F. japonica* and 10 clones of *F. sachalinensis*, that is, a total of 50 clones. These clones had been collected across a latitudinal gradient from southern Switzerland to northern Germany, and their taxon identities confirmed through molecular markers (Krebs et al. 2010). For *Fallopia × bohemica* and *F. sachalinensis*, every clone used in our experiment is a unique genotype, whereas all *F. japonica* clones are genetically identical (Krebs et al. 2010), as appears to be the case for all *F. japonica* in Europe (Hollingsworth and Bailey 2000; Mandak et al. 2005). Prior to our experiment, all clones were cultivated under identical conditions for several years in the Botanical Garden of Marburg, Germany. For the native plant community, we selected several species that commonly occur in the habitats invaded by knotweed (Gerber et al. 2008): *Geranium robertianum*,*Geum urbanum*,*Silene dioica, Symphytum officinale* and *Urtica dioica*. We used seed material from a regional supplier of wild-collected seeds (Rieger-Hofmann GmbH, Blaufelden-Raboldshausen, Germany).

### Experimental set-up

In August 2010, we filled 500 4-L pots with a mixture of 1:1 sand and field soil (RICOTER Erdaufbereitung AG, Aarberg, Switzerland). In each pot, we planted the same native plant community, with one seedling of each of the native species, and seedlings arranged in a circle. After 10 days, when the native seedlings had successfully established, we planted one 8-to 10-cm piece of knotweed rhizome, with two intact nodes, in the centre of each pot, at 5 cm below the soil surface. Prior to planting, we measured the length and diameter of each of the planted rhizomes.

We planted 10 replicates of each clone, a total of 500 pots. To test for potential allelopathic effects of invaders, we added activated carbon (Charcoal Activated, Merck KGA, Darmstadt, Germany) at a concentration of 20 mL/L soil to half of the pots. Activated carbon (AC) is often used to test for the presence of allelochemicals in the soil because of its high capacity to adsorb organic compounds (e.g. Callaway and Aschehoug 2000; Inderjit and Callaway 2003; Prati and Bossdorf 2004). Although AC can sometimes have direct effects on plant growth (Lau et al. 2008) and interfere with plant–soil biota interactions (Weisshuhn and Prati 2009), it remains a very useful, simple tool for experimental tests of allelopathic effects. The main experiment took place in an experimental garden in Muri b. Bern, Switzerland. The pots were placed in a large garden bed in fully randomized order. Three weeks after the set-up, we measured the sizes of all native plants and recorded the emergence of knotweed shoots aboveground as a measure of regeneration success. To avoid nutrient depletion during the experiment, we applied liquid NPK fertilizer (7:5:6) equivalent to 30 kg N/ha once in April 2011. In August 2011, we separately harvested the aboveground biomass of each species in each pot, dried it at 80°C for 72 h and weighed it.

### Statistical analyses

We analysed differences in knotweed regeneration success using a generalized linear model with binomial error that included the main effects of taxon (3 levels), clone nested within taxon (50 levels), activated carbon (2 levels) and the interactions. We further explored differences among and within taxa through a series of contrasts comparing hybrids to their parents, comparing the two parental taxa, as well as testing the significance of within-taxon clone variation separately for each taxon (see Table 1). For the log-transformed knotweed biomass, we fitted a linear model with the same model structure as above.

**Table 1:** The effect of knotweed taxon and clone identity, as well as addition of activated carbon, on knotweed performance and growth of a native plant community. We use contrasts to further partition the variance explained by taxon and clone effects. Significant effects are shown in bold.

	Knotweed regeneration			Knotweed biomass				Native biomass			
Source of variation	d.f.	*χ*²	*P*-value	d.f.	SS	*F*	*P*-value	d.f.	SS	*F*	*P*-value
Taxon	2	0.47	0.790	2	30.5	10.90	**<0.001**	2	240	3.05	**0.049**
Hybrid vs. Parents	1	0.46	0.496	1	30.4	16.07	**<0.001**	1	238	6.06	**0.015**
*F. japonica* vs. *F. sachalinensis*	1	0.01	0.922	1	0.0	0.01	0.931	1	2	0.05	0.821
Clone	47	140.46	**<0.001**	44	83.4	2.35	**<0.001**	45	3442	1.95	**0.001**
*F. japonica* clones	12	35.03	**<0.001**	12	22.2	2.29	**0.010**	12	316	0.67	0.779
*F. sachalinensis* clones	9	35.51	**<0.001**	7	7.2	1.27	0.267	7	1513	5.51	**<0.001**
*F. × bohemica* clones	25	69.92	**<0.001**	25	54.0	2.68	**<0.001**	26	16131	1.58	**0.045**
AC	1	14.08	**<0.001**	1	1.4	1.73	0.190	1	1164	29.65	**<0.001**
Taxon: AC	2	12.45	**0.002**	2	0.9	0.66	0.523	2	42	0.54	0.334
Clone: AC	46	69.61	**0.014**	37	24.2	0.81	0.771	37	1597	1.10	0.334
Residual	402			178	143.6			178	6989		

To analyse the impact of invasive knotweed on native plant communities, we first used a simple *t*-test to compare the total native biomass in the 310 pots where knotweed was present to the 190 pots where it was not present at the end of the experiment, either because rhizomes did not regenerate or because plants had died during the experiment. As a second step, we analysed the total native biomass from all pots where knotweed was present using a linear model that tested the effects of knotweed taxa, clones nested within taxa, AC and their interactions, with the same contrasts as above.

To account for possible effects of initial size differences between knotweed rhizomes and native plant seedlings, we initially included the volumes of planted knotweed rhizomes and the sizes of native plant seedlings as covariates in the analyses. However, these covariates were never significant, and we therefore eventually dropped them from our analyses.

## Results

Most of the native plants survived transplanting, and 95% of them were present at the end of the experiment. However, only about two-third of the planted knotweed rhizomes regenerated during the course of the experiment, and about 80% of those plants were still present at the time of harvesting, which reduced the final size of the experiment for analysing taxon and clone biomass differences by about half. In pots where it regenerated, knotweed became one of the dominant species and constituted on average more than a quarter of the biomass. Among the natives, *Silene dioica* was the most successful species and accounted for about half of the native biomass. The second most successful native species was *Symphytum officinale*, followed by *Geranium robertianum*,*Urtica dioica* and *Geum urbanum*.

**Figure 1:**
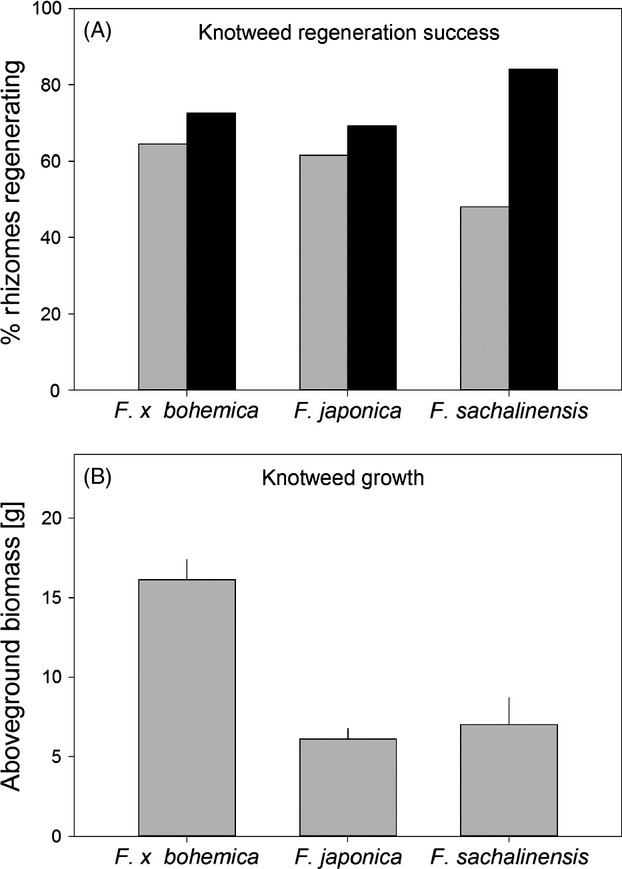
Regeneration success (A) and growth (B) of invasive knotweed hybrids and their parents when grown in an experimental community of five native plant species. In panel A, the black bars are with activated carbon added to the soil, and the grey bars are without.

**Figure 2:**
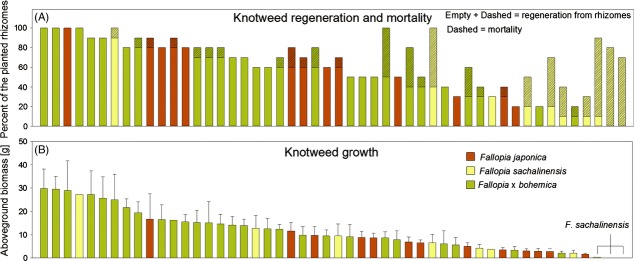
Regeneration, mortality (A), and average growth (B) of the 50 studied knotweed clones. Rhizomes of each clone were planted in a community of native species, with 10 replicates per clone. Regeneration from rhizomes was recorded 2 weeks after planting, whereas mortality was assessed 1 year later, at the time of biomass harvesting.

The average rate of regeneration of rhizomes was very similar among the three knotweed taxa (Table 1, Fig. 1A). Addition of activated carbon generally improved knotweed regeneration, and this effect was particularly strong for *F. sachalinensis*, where addition of AC increased regeneration by more than 50% (Fig. 1A). There was significant variation in regeneration rates among clones of all three taxa (Table 1, Fig. 2A), and there were also significant differences in the responses of different clones to AC.

We found a highly significant taxon effect for knotweed biomass. This effect was entirely driven by the significant difference between the hybrid *Fallopia* × *bohemica* which grew on average three times better that its parents (hybrid vs. parents contrast in Table 1, Fig. 1B), whereas the parental species *F. japonica* and *F. sachalinensis* did not differ in their biomass. There was significant intraspecific variation in biomass among the clones of *F. × bohemica* and *F. japonica*, but not among those of *F. sachalinensis* (Table 1, Fig 2B). Addition of AC had no effect on knotweed biomass.

In pots where knotweed was present, the average total biomass of the native community was 20% lower than in pots were knotweed did not regenerate or where it died during the experiment (*t *=* *1.83, *P *=* *0.043). Analysis of the pots where knotweed was present showed that the hybrid had a stronger negative impact on native plants than its parents (Table 1). There was significant variation in the impact of different *F. sachalinensis* and *F. × bohemica* clones. Addition of AC significantly decreased total native biomass by 15%.

## Discussion

Hybridization can contribute to invasion success if hybrids perform better or are more competitive than their parents. Here, we found that *Fallopia* × *bohemica*, the hybrid between the two invasive knotweeds, *F. japonica* and *F. sachalinensis*, performed substantially better in experimental communities of native plants, and it had a greater impact on the biomass of the native plants. Our study confirms that invasive knotweed hybrids are indeed even more competitive than their parents and that hybridization significantly increased the invasiveness of the exotic knotweed complex.

### Differences among taxa

Regeneration from rhizomes is one of the main ways by which knotweed spreads in its invasive range. The specific measure of regeneration success we used in our study – the presence of young shoots aboveground – could be influenced by several factors: the numbers of buds on the planted rhizomes, the ability of plants to activate these buds or to produce new buds, and the resistance of the buds and new shoots to soil-borne pathogens and herbivores. In our experiment, we did not discriminate between these individual factors but rather integrated across them.

We found that rates of regeneration from rhizomes were generally similar between knotweed hybrids and their parents. Only in the control soils, the rhizomes of *F. sachalinensis* regenerated less well. It is unclear whether this is an inherent characteristic of the giant knotweed or a result of its lower investment into chemical defences (Krebs et al. 2011), because addition of activated carbon eliminated these differences in our experiment. This suggests that some kind of root exudates must be involved, either exudates of *F. sachalinensis* that stimulate the activity of soil pathogens or exudates of other species with similar effects. In any case, the observed reduced regeneration success of *F. sachalinensis*, which has also been found in other studies (Bimova et al. 2003), may be one of the reasons why the species – in spite of its spectacular growth – is generally less widespread in its introduced range (Mandak et al. 2004).

In spite of their similar overall regeneration rate, knotweed hybrids that grew in a community of native plants had almost three-fold final biomass compared with their parents. This increased performance is likely reflecting superior competitive ability and thus an important potential determinant of invasion success. Our results could thus explain why knotweed hybrids spread faster than their parents in the field (Mandak et al. 2004). The underlying mechanism of this increased performance could be a more efficient use of resources, as indicated by previous studies (Aguilera et al. 2010; Dassonville et al. 2011), or it could be increased allelopathy. In both cases, the superior attributes of the hybrid may either result from a heterosis effect or from the expression of genetic material that is unique to the hybrid (Krebs et al. 2010), possibly reflecting parental genetic material not introduced to Europe.

One possibility for explaining greater dominance could be increased allelopathic potential of the hybrids. Several previous studies found allelopathic effects of invasive knotweeds on native plants (Siemens and Blossey 2007; Murrell et al. 2011). In our study, we tested for allelopathy through addition of activated carbon, and we compared how this affects the success of knotweed hybrids versus their parents. However, we found hardly any evidence for differences among the knotweed taxa, and in fact, no evidence for allelopathy at all. On the contrary, addition of activated carbon had a positive effect on knotweed and negative effects on the natives, which indicates allelopathic effects of the natives on knotweed, rather than vice versa. Together with one of our previous experiments (Parepa et al. 2012), our results suggest that knotweed allelopathy, if it exists at all, might play a role in later stages of the invasion process, but not during the early phase of knotweed establishment.

Of course, our results may be to some degree contingent upon the specific set-up that we chose for our experiment and the native species that we chose as competitors. We established native communities from seedlings, but knotweed was introduced through rhizomes. With this, we attempted to simulate a situation where knotweed fragments are thrown or washed into ruderal-riparian communities, which is a realistic and common situation for knotweed invasion. We did not test the invasion of knotweed into taller, more established communities or its establishment from seed. For the native competitors, we selected five of the most common native species known to successfully co-occur with knotweed (Krebs et al. 2010). Several of these are strong competitors themselves and reported as invaders on other continents (Randall 2002), so our experiment was probably a rather rigorous one, and its results should be valid mostly for the nutrient-rich plant communities in which our native species usually occur.

### Variation within taxa

As our experiment included multiple clones of the three different knotweed taxa, it allowed us to explore their variability in invasion success. We found that there was substantial within-taxon variation in the hybrids and in the two parental species. Hybrid clones differed in regeneration, growth and impact, and there was also great variation in regeneration and impact among different clones of *F. sachalinensis*, as well as variation in regeneration and growth, but not impact, among different clones of *F. japonica*. For *F. sachalinensis* and *F. × bohemica,* these different clones represent distinct genotypes (Krebs et al. 2010), but for *F. japonica,* they do not. Not only the clones of *F. japonica* used in our study, but apparently all invasive populations in Europe are genetically uniform (Hollingsworth and Bailey 2000; Mandak et al. 2004; Krebs et al. 2010).

Several previous studies (Tiebre et al. 2007; Krebs et al. 2010) also analysed the genetic diversity of knotweed hybrids within and across multiple populations in Europe and demonstrated that at the molecular level, the hybrid generally has a composition intermediate between the two parents, but it also contains a characteristic unique gene pool. Here, we can confirm that the molecular variability is matched by a corresponding phenotypic variability (see also Herpigny et al. 2012 for a study under field conditions). It is likely that the variation within all invasive knotweed taxa increases their ecological amplitude and thereby contributes to their invasion success.

Clones of *F. japonica* are genetically identical and should therefore be expected to lack heritable phenotypic variation. However, we find heritable variation among clones, and it is an intriguing question what is causing this variation. One possibility could be carry-over effects of environmental differences between the habitats where the different clones have originally been collected. However, prior to our experiment, the clones we used had been precultivated for several years, which makes this explanation rather unlikely. Another possibility could be that the genetically identical *F. japonica* clones harbour epigenetic variation. There is increasing evidence that natural epigenetic variation is common within and among plant populations, and that epigenetic variation can cause significant phenotypic variation (Bossdorf et al. 2008; Gao et al. 2010; Herrera and Bazaga 2010, 2011). There is some evidence that different, genetically identical clones of *F. japonica* can indeed be epigenetically variable (Richards et al. 2012; Y. Zhang & O. Bossdorf, unpublished data). However, the causal link between this epigenetic variation and the observed phenotypic variation remains yet to be demonstrated.

If *F. japonica* is indeed epigenetically variable, and this variability plays a role in invasion success, then this could also play a role in *F. × bohemica*, because epigenetic variability will be amplified in the hybrid. Epigenetic processes are known to play a key role in hybridization events (Wendel 2000), for example through stabilizing novel hybrid genotypes (Finnegan 2002), and they could contribute to the rapid radiation and adaptation of invasive hybrid taxa (Prentis et al. 2008). Several previous studies showed that invasive hybrids and polyploids undergo massive epigenetic rearrangements and speculated about the role of these epigenetic changes for their invasion success (e.g. Salmon et al. 2005; Parisod et al. 2009). More research is needed to explore the causal relationships between epigenetic variation, phenotypic variation and competitive dominance across different invasive knotweed taxa.

In summary, our experiment indicates that hybrid knotweed is more successful at invading native plant communities and that it harbours more phenotypic variation than either of its parents. Our study thus confirms previous field studies which found knotweed hybrids to be more successful and faster spreading than their parents (Pysek et al. 2003, 2009; Mandak et al. 2004). More generally, our study shows that a recent hybridization between two introduced species has created a novel and even more invasive taxon, and it adds to the increasing evidence that hybridization is an important mechanism in many plant invasions (Abbott 1992; Ellstrand and Schierenbeck 2000; Zalapa et al. 2010; Blair et al. 2012). From a practical point of view, our study suggests that it may be advisable to prioritize management efforts on knotweed hybrids, not only because they can, unlike *F. japonica*, produce viable seeds and in addition act as a pollen source for the single female *F. japonica* clone, but also because the hybrids are inherently the most vigorous of the invasive knotweeds.

## Data Archiving Statement

Raw data for this study are available from the Dryad Digital Repository: http://dx.doi.org/10.5061/dryad.hr768.
